# Acknowledgment to Reviewers of *Antibodies* in 2020

**DOI:** 10.3390/antib10010005

**Published:** 2021-01-25

**Authors:** Antibodies Editorial Office

**Affiliations:** MDPI AG, St. Alban-Anlage 66, 4052 Basel, Switzerland; antibodies@mdpi.com

Peer review is the driving force of journal development, and reviewers are gatekeepers who ensure that *Antibodies* maintains its standards for the high quality of its published papers. Thanks to the cooperation of our reviewers, in 2020, the median time to first decision was 17 days and the median time to publication was 59.5 days. The editors would like to express their sincere gratitude to the following reviewers for their precious time and dedication, regardless of whether the papers were finally published:
Adolf-Bryfogle, JaredLiu, XiangleiÅhlin, AndersLo, Shyh-ChyiAlam, Md KausarLopalco, LuciaAlbarnaz, Jonas D.Lub-de Hooge, Marjolijn N.Al-Saleem, FetwehLucas, Andrew T.Alt, KarenMa, BuyongAngkawidjaja, ClementMack, MatthiasAntigua, Khristine Joy C.Maglio, OrnellaArunkumar, AbhiramManez, RafaelAxiotis, EvangelosManna, SaikatAzam, Aa HaerumanMansour, SalahBan, BhupalMartin, Andrew C. R.Barfield, RobynMartins, SoniaBawadekar, MandarMatsushita, ShuzoBax, HeatherMazor, OhadBernard, NicoleMehta, ParasBerrondo, MonicaMo, FeiBerthelot, LaurelineMukherjee, ShibabrataBianchini, RodolfoMukherjee, SomnathBlot-Chabaud, MarcelNemenoff, RaphaelBoyd, AndrewNesterov-Mueller, AlexanderBreitling, FrankNicoletti, LoredanaBrooks, Cory L.Nijhof, IngerBulatov, EmilNishikimi, AkihikoChalla, DilipNordenfelt, PontusChen, LongOh, Eun-JeeChen, Shih-ChiehOnaca, NicholasCheng, Luisa W.Ortiz, AndricDahiya, SatinderOzen, MehmetDanilov, Sergei M.Pasternak, GerardDar, Mohd SaleemPatil, VeerupaxagoudaDavey, MartinPellefigues, ChristopheDe Kruif, JohnPezzoni, GiuliaDebadyuti, GhoshPlans-Rubi, PedroDessain, Scott K.Poole, Brian D.Devasundaram, SanthiProfumo, ElisabettaDevaurs, DidierQi, JunpengDixit, SurjitRader, ChristophDuranti, ClaudiaRavichandran, SupriyaEason, JamesReddy, Sai T.Elvington, MichelleReuter, StefanEscobar-Cabrera, EricRieder, MichaelFuentes-Lopez, SandraRiley, LeeGallagher, D. TravisRispens, TheoGan, SamuelRokstad, Anne MariGanguly, SamitRoumenina, LubkaGarcia Parra, JezabelRubin, Robert L.Gilburd, BorisRusso, GiulioGolay, JoseeSasaki, MotokoGorovits, BorisScheu, StefanieGorshkova, Ekaterina N.Schlothauer, TilmanGosav, Evelina MariaSchorzman, Allison N.Gottschalk, StephenSchrum, AdamGoulet, Dennis R.Scott, AndrewGrubić Kezele, TanjaSharma, PreetiHadži, SanShim, HyunboHalkias, JoannaSochaj-Gregorczyk, Alicja MariaHamilton, Robert G.Spoerl, DavidHammers, Christoph M.Stang, EspenHatano, YuichiroStrasser, RichardHazlett, KarstenSun, WeiHealey, GarethSutton, BrianHiruta, YukiTakagi, MotokiHoebeke, JohanTanha, JamshidHomma, TakujiroTeplyakov, AlexeyHouen, GunnarThaker, Youg RajHwang, DobeenTheise, NeilHytti, MariaTsuchikama, KyojiIaccino, EnricoVance, DavidIdda, Maria LauraVarki, Nissi M.Ippolito, GregoryVartak, AbhishekJakobsson, EricVattepu, RaviJayaraman, SundararajanVelikova, TsvetelinaJeliazkov, Jeliazko R.Venkatesan, ThiagarajanJohnson, Dylan M.Vidarsson, GesturJones, MartinaWagner, AlainJoshi, KamalWang, ShaobinKalra, Raj SinghWeitkamp, HendrikKang, Min-JungWheatley, AdamKarsten, Christina BeatriceWilliamson, E. DianeKazemian, MajidWu, Yee LingKhan, NaeemXiao, YongshengKhatun, AminaYamaki, KouyaKolawole, AbimbolaYang, KunKorona, DagmaraYin, TianleiKowal, KrzysztofYu, ChunsongKrause, FrankZagożdżon, RadosławLamichhane, PurushottamZhang, RuiLee, SukmookZhang, WeipingLeyton, Jeffrey V.Zhang, XiangminLi, TommyZhou, QunLima, Wanessa C.Zhou, WeijieLiou, Chian-JiunZielonka, Stefan

